# Methodology for Large-Scale Camera Positioning to Enable Intelligent Self-Configuration

**DOI:** 10.3390/s22155806

**Published:** 2022-08-03

**Authors:** Yingfeng Wu, Weiwei Zhao, Jifa Zhang

**Affiliations:** School of Mechanical and Electronic Engineering, Wuhan University of Technology, 122 Luoshi Road, Wuhan 430070, China; wyf1997@whut.edu.cn (Y.W.); zjf94131@whut.edu.cn (J.Z.)

**Keywords:** large-scale positioning and navigation, intelligent self-configuration, collaborative visual network, extended Kalman filter

## Abstract

The development of a self-configuring method for efficiently locating moving targets indoors could enable extraordinary advances in the control of industrial automatic production equipment. Being interactively connected, cameras that constitute a network represent a promising visual system for wireless positioning, with the ultimate goal of replacing or enhancing conventional sensors. Developing a highly efficient algorithm for collaborating cameras in the network is of particular interest. This paper presents an intelligent positioning system, which is capable of integrating visual information, obtained by large quantities of cameras, through self-configuration. The use of the extended Kalman filter predicts the position, velocity, acceleration and jerk (the third derivative of position) in the moving target. As a result, the camera-network-based visual positioning system is capable of locating a moving target with high precision: relative errors for positional parameters are all smaller than 10%; relative errors for linear velocities (*v_x_*, *v_y_*) are also kept to an acceptable level, i.e., lower than 20%. This presents the outstanding potential of this visual positioning system to assist in the industry of automation, including wireless intelligent control, high-precision indoor positioning, and navigation.

## 1. Introduction

Large-scale camera positioning systems, also known as visual positioning systems, are used for precisely locating or tracking a moving object in three-dimensional space. Compared with other positioning technologies, e.g., laser-tracking, theodolite, RFID, geomagnetic field, or indoor GPS (iGPS), visual positioning systems have received intense research interest because of advantages such as low cost, high precision, and contactless tracking functions [1,2,3].

Therefore, they have wide application prospects in various industrial areas, such as motion analysis, robot guidance, or aircraft manufacturing [4,5,6,7]. According to the literature, visual positioning systems can be categorized into two types, as defined by the number of cameras: single pan-tilt-zoom (PTZ) camera, and camera network [8]. Due to the superiorities of locating accuracy, field of view, and response speed, positioning systems based on camera networks are regarded as a more promising system type than those based on a single PTZ camera [9].

Positioning systems based on camera networks are able to adjust the visual scale, by choosing appropriate numbers of cameras, to meet various production requirements. Galetto et al. presented a visual positioning system called MScMS-II, which was able to track two targets in a cubic space with length of 2 m [10]. The coordinates of the targets could be calculated using data acquired from at least two cameras. Dixon et al. successfully tracked vehicles in an urban environment by setting up seven cameras [11]. A system using the interaction of 30 cameras was set up by Wei et al. for monitoring an area of 1670 m^2^ [12]. Approximately 100 stationary cameras were integrated by Kuo et al. [13], enabling a high degree of collaborative working among the cameras, for campus monitoring. Jiarui Lin et al. presented a ceiling-mounted workshop measurement positioning system (C-wMPS), to compensate for many deficiencies shown by conventional metrology systems [14]. Pablo Puerto et al. designed a methodology to evaluate the measurement uncertainty of portable photogrammetry for LVM [15]. Domenico Augusto Maisano et al. presented some diagnostic tests for combinations of LVM systems that are equipped with distance and/or angular sensors [16]. Yongkang Lu et al. developed a novel nonlinear optimization method to reduce the overall calibration error of the LRS [3]. Apparently, then, the number of cameras is varied according to the corresponding application requirements. Effective management of the cameras is able to improve the locating accuracy, increase the responding speed, and minimize the energy consumption. Soro et al. developed two methods for choosing the cameras’ visual area, aiming to avoid redundant data and reduce the processing time [17]. The research group of Liu et al. introduced a dynamic collaborative scheme for tracking moving targets, based on wireless camera networks [18]: sequential Monte Carlo (SMC) method, i.e., particle filtering, was utilized for calculating the probability of detecting a target and assigning the camera with the highest probability as the chief camera from the cluster. Another particle filter, based on color, was investigated by Liang et al. for switching cameras within the network [9]. They achieved a significant reduction in redundant video data, and enabled the simultaneous location of five targets, in real time, using an ordinary personal computer.

We proposed an update to the system, by software synchronization method, and formulated the solution for the synchronization of cameras. The mean value of standard deviation of eight cameras, mounted on two workstations, was 12.53 ms, the localization performance of LSVLS was enhanced [19]. We optimized the cameras’ placement using a relative positioning algorithm (RPA). The result was that optimal camera placement greatly enhances the efficiency of camera placement in LSVLS, as verified with a field-winding mobile vehicle model [20].

In this work, a visual positioning system, based on a camera network consisting of 16 cameras, was built for locating a filament winder. A novel strategy for: selecting cameras; assigning a chief camera; and conducting collaborative data transfer, which enables intelligent self-configuration of the camera network; was developed and described in detail. The feasibility and reliability of the positioning system, in terms of the locating method and predicting algorithm, was experimentally demonstrated, i.e., prediction of the position and velocity of the filament winder.

## 2. Methodology

### 2.1. Positioning System

**Figure 1:**
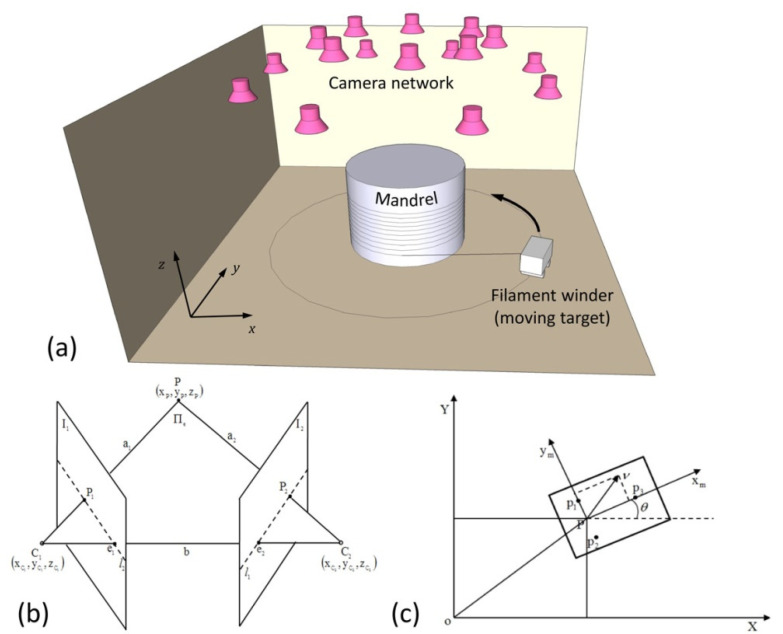
(**a**) Schematic diagram for the experimental setup in this work. A total of 16 cameras constitute a camera network for the visual positioning system. (**b**) Graphic illustration of the principle of stereo vision. (**c**) Method of locating the moving target (filament winder).

A filament winder was recognized as the moving target in this work. A mandrel with a diameter of 5 m was located at the center of the working space (see Figure 1a). The filament winder moved around the mandrel, as the default trajectory, with a constant speed. The circular motion of the filament winder was attended by a slight ascending motion of the mandrel, with the aim of avoiding overlap of the fiber during winding. As is illustrated in Figure 1a, 16 cameras were installed above the working space, directly above the circular trace of the filament winder. The visual positioning system was thereby established, with these 16 cameras, to navigate the filament winder while the winding operation took place. The location of the cameras in the visual positioning system, in this work, were designed following guidelines from the literature [19,20].

A commercial laptop (Lenovo notebook, equipped with 1.9 GHz CPU and 2 GB memory) was used as the hardware for data-processing in the visual positioning system. Cameras used in this work, which constitute the camera network, were infrared cameras from Nintendo Co., Ltd., Kyoto, Japan (also known as Wiimote, IR camera), with 1024 × 768 pixels of interpolated resolution, 45° × 30° field of vision, and 100 Hz sampling rate. The real-time connection between the laptop and the cameras was enabled by long-distance Bluetooth modules. The remotely controllable filament winder was wirelessly connected with the laptop, and navigated via instructions from the laptop.

#### 2.1.1. Stereo Vision

The function attribute of the positioning of the moving target was stereo vision, which was obtained by at least one camera. Figure 1b illustrates a simplified model for establishing stereo vision on one target (point *P*) by two cameras (*C*_1_ and *C*_2_). As shown in Figure 1b, the images of the target acquired by the cameras are marked as *P*_1_ on plane *I*_1_, and *P*_2_ on plane *I*_2_, corresponding to the two cameras *C*_1_ and *C*_2_, respectively. Thus, the coordinates of *P*_1_ and *P*_2_, i.e., *x*_1_ or *x*_2_, are given by [21]:

(1) x=CX

where *X* denotes the coordinates of target *P*, *C* is the projective matrix of cameras.

Therefore, the bunch of divergence lines passing through the center of the cameras and the target area can be expressed in Equation (2):

(2) $$X\left(\mu \right)=\mu \left(\begin{array}{c} M^{-1}x\\ 0 \end{array}\right)+\left(\begin{array}{c} -M^{-1}p4\\ 1 \end{array}\right)=\left(\begin{array}{c} M^{-1}\left(\mu x-p4\right)\\ 1 \end{array}\right)$$

where, *M =* *C* (1:3, 1:3), *p*4 = *C* (:,4). –*M*^−1^*p*4 denotes the camera center, and *M*^−1^*x* gives the direction of the divergence lines. *a*_1_ and *a*_2_ connect the target and the cameras, which stand for the central visual paths. By intersecting these central visual paths with the image planes (*I*_1_ and *I*_2_), the coordinate of the target *P* can, thereby, be calculated, using Ray 3D in robotics and machine vision toolboxes [22].

#### 2.1.2. Methodology of Locating the Moving Target (the Filament Winder)

According to the experimental setup in this work, the filament winder, as the tracking target, moved around the mandrel, while the mandrel lifted slightly, with a constant speed, to complete the winding process. Thus, the circular motion of the filament winder was the primary dynamic motion, which had to be navigated. Based on conventional locating techniques reported in the literature [23,24], the coordinates (*x*, *y*, *z*) and orientation (*θ*) of the moving target in this work can be described as:

(3) $$q={\left[x\;y\;z\;\theta \right]}^{T}$$

As is illustrated in Figure 1c, three feature points on the moving target were chosen and marked as *p*_1_*, p*_2_, and *p_3_*. Point *P*, shown in Figure 1b, is the midpoint of *p*_1_ and *p*_2_, having a location point at the rear of the filament winder. Point *p_3_* is located on the front of the filament winder. The line connecting *P* and *p_3_* is vertical to the line *p*_1_ to *p*_2_. The angle between the line *P-p_3_* and the *X* axis denotes the orientation *θ* of the moving target. Because the coordinates of *p*_1_*, p*_2_, and *p_3_* can be precisely acquired by the camera-network-based visual positioning system, the position and orientation of the moving target can be calculated by using Equations (4) and (5) as follows:

(4) $$P=\frac{1}{\;2}\left(p_{1}+p_{2}\right)$$

(5) $$\theta =arctan2\left(y_{p3}-y_{P},\;x_{p3}-x_{P}\right)$$

### 2.2. Data Processing Using Extended Kalman Filter

Kalman filter is known as a linear, discrete time, finite-dimensional time-varying system, used to evaluate estimates for the state variables and minimize the mean-square error [24]. A non-optimal approach to solving the problems with non-Gaussian functions, in the frame of linear filters, is the extended Kalman filter (EKF). The EKF implements a Kalman filter for system dynamics that results from the linearization of the original nonlinear filter dynamics around the previous state-variable estimates [25,26,27]. In this work, EKF was conducted to cope with the prediction of velocity, acceleration, and jerk (the third derivative of position) in the moving target.

According to the Equation (3), the velocity can be derived by taking the derivative with respect to the position *q*, which is given by $v_{x}=\dot{x},\;v_{y}=\dot{y},\;w=\dot{\theta }$. Similarly, the acceleration and jerk can be obtained by taking two or three times the derivative to the position *q*, respectively. Thus, the expressions for velocity, acceleration, and jerk of the moving target are listed in Equation (6):

(6) $$\{\begin{array}{c} \dot{q}={\left[v_{x}\;v_{y}\;w\right]}^{T}\\ \ddot{q}={\left[\dot{v_{x}}\;\dot{v_{y}}\;\dot{w}\right]}^{T}\\ \dddot{q}={\left[\ddot{v_{x}}\;\ddot{v_{y}}\;\ddot{w}\right]}^{T} \end{array}$$

These differential operations, applied to the position, require the discretization of the moving target model when conducting the EKF calculation. The discretization of the model in terms of position, velocity, acceleration, and jerk is listed in Equation (7):

(7) $$\{\begin{array}{c} x_{k}=x_{k-1}+Ts*v_{x\left(k-1\right)}\\ y_{k}=y_{k-1}+Ts*v_{y\left(k-1\right)}\\ z_{k}=z_{k-1}+Ts*v_{z\left(k-1\right)}\\ \theta_{k}=\theta_{k-1}+Ts*{\dot{\theta }}_{\left(k-1\right)}\\ v_{xk}=v_{x\left(k-1\right)}+Ts*{\dot{v}}_{x\left(k-1\right)}\\ v_{yk}=v_{y\left(k-1\right)}+Ts*{\dot{v}}_{y\left(k-1\right)}\\ v_{zk}=v_{z\left(k-1\right)}+Ts*{\dot{v}}_{z\left(k-1\right)}\\ {\dot{\theta }}_{k}={\dot{\theta }}_{k-1}+Ts*{\ddot{\theta }}_{\left(k-1\right)}\\ {\dot{v}}_{xk}={\dot{v}}_{x\left(k-1\right)}+Ts*{\ddot{v}}_{x\left(k-1\right)}\\ {\dot{v}}_{yk}={\dot{v}}_{y\left(k-1\right)}+Ts*{\ddot{v}}_{y\left(k-1\right)}\\ {\dot{v}}_{zk}={\dot{v}}_{z\left(k-1\right)}+Ts*{\ddot{v}}_{z\left(k-1\right)}\\ {\ddot{\theta }}_{k}={\ddot{\theta }}_{k-1}+Ts*{\dddot{\theta }}_{\left(k-1\right)}\\ {\ddot{v}}_{xk}={\ddot{v}}_{x\left(k-1\right)}\\ {\ddot{v}}_{yk}={\ddot{v}}_{y\left(k-1\right)}\\ {\ddot{v}}_{zk}={\ddot{v}}_{z\left(k-1\right)}\\ {\dddot{\theta }}_{k}={\dddot{\theta }}_{\left(k-1\right)} \end{array}$$

In prior to the EKF calculation, measured input ${\tilde{y}}_{k}$, and the variable to be solved in terms of state vector $x_{k}$, have been pre-described and given by:

(8) $$x_{k}=\left[\hat{x}\;\hat{y}\;\hat{z}\;\hat{\theta }\;{\hat{v}}_{x}\;{\hat{v}}_{y}\;{\hat{v}}_{z}\;\hat{\dot{\theta }}\right]$$

(9) $${\tilde{y}}_{k}=\left[p_{1}\;p_{2}\;p_{3}\right]$$

When conducting the EKF calculation, two Jacobians (*F* and *H*) are required for the predictor-corrector algorithm. These two Jacobians are defined by:

(10) $$F=\left[\begin{array}{cccc} I_{4\times 4} & Ts\cdot I_{4\times 4} & 0_{4\times 4} & 0_{4\times 4}\\ 0_{4\times 4} & 0_{4\times 4} & Ts\cdot I_{4\times 4} & 0_{4\times 4}\\ 0_{4\times 4} & 0_{4\times 4} & 0_{4\times 4} & Ts\cdot I_{4\times 4}\\ 0_{4\times 4} & 0_{4\times 4} & 0_{4\times 4} & I_{4\times 4} \end{array}\right]\;$$

(11) $$H=\left[\begin{array}{cc} I_{4\times 4} & 0_{4\times 12} \end{array}\right]$$

In the extended-Kalman filter, mathematically, the predictor step is given by:

$${\hat{x}}_{k}^{-}=\;f\left({\hat{x}}_{k-1},u_{k},k\right)$$

$$P_{k}^{-}={\;F}_{k-1}P_{k-1}F_{k-1}^{T}+Q_{k}$$

(12) $$K_{k}=P_{k}^{-}K_{k}^{T}{\left(H_{k}P_{k}^{-}H_{k}^{T}+R_{k}\right)}^{-1}$$

$${\hat{x}}_{k\;}={\hat{x}}_{k}^{-}+K_{k}\left({\tilde{y}}_{k}-\;h\left({\hat{x}}_{k}^{-},u_{k},k\right)\right)$$

$$P_{k}=\left(I\;-K_{k}H_{k}\right)P_{k}^{-}$$

In the above equations $P_{k}$ is an estimate of the covariance of the measurement error and $K_{k}$ is called the Kalman gain. After both the prediction and correction steps have been performed, then ${\hat{x}}_{k}$ is the current estimate of the states and ${\hat{y}}_{k}$ can be calculated directly from it.

## 3. Experimental Approaches

### 3.1. Calibration of Cameras

**Figure 2:**
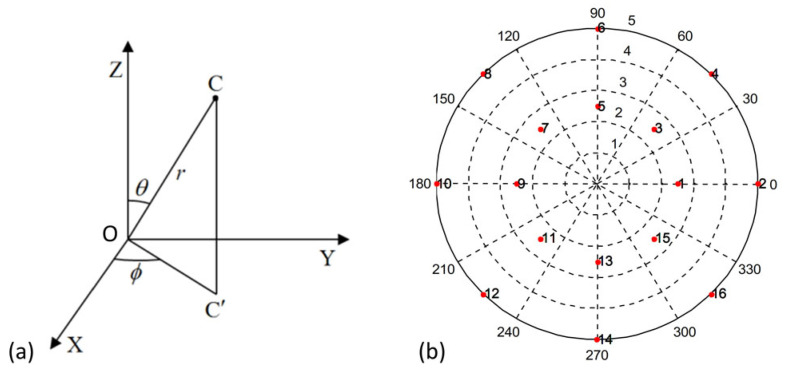
(**a**) The location of Camera *C* can be described by three parameters *r, φ, θ* in three-dimensional space. (**b**) The optimal arrangement of the 16 cameras for the current investigation. Cameras were arranged as two circles surrounding the center of the mandrel, with even distribution. Each circle consisted of 8 cameras.

According to the fundamental research of our group [19,20], a relative algorithm, based on integer linear programming, was proposed. This algorithm was utilized in this work to optimize the placement of the cameras, in terms of determining the optimal position for the cameras individually, with the aim of saving processing time, reducing the computational consumption, and guaranteeing the reliability of the positioning system in controlling the filament winder. The algorithm was inserted and programmed in the Multi-Camera Self-Calibration Toolbox [28] for practical initiation of the cameras in this work. As illustrated in Figure 2a, the position of the cameras in the working space was quantitatively assigned using three parameters: distance of camera *C* to the origin of the reference coordinate *O* (*r*); the angle between the *X* axis and the projection line *OC’* (*φ*); and the angle between the *Z* axis and line *OC* (*θ*). Hence, the calibration parameters for cameras, i.e., position and orientation, were precisely determined and imported into the visual positioning system, thereby achieving the optimal initial boundary conditions. The optimum organization of the 16 cameras is demonstrated in Figure 2b. Because of the circular default trajectory of the filament winder, the cameras were organized as two circles around the center of the mandrel. Each circle contained 8 cameras, in order to guarantee that at least two cameras were able to locate the filament winder at any point in the designated trajectory.

### 3.2. Algorithm for Automatically Selecting Cameras

**Figure 3:**
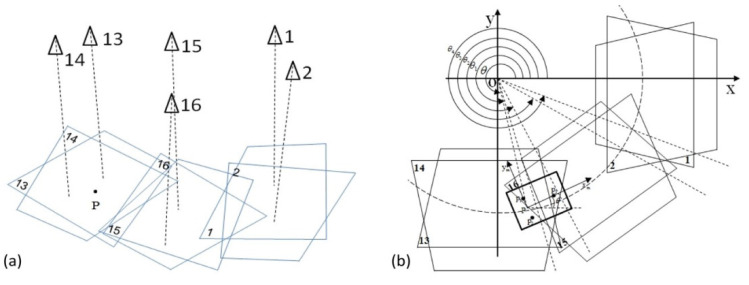
(**a**) Schematic diagram of the rectangular field of visions, which are projected on the horizontal plane (*XY* plane), corresponding to the cameras with numbers 1, 2, 13, 14, 15, 16. (**b**) The moment of moving target (*p*) leaving the rectangular fields of vision of cameras 13 and 14, and entering into the fields of vision of cameras 15 and 16. The parameter *θ*, the angle between *X* axis and the line *OP*, is the numerical criterion for selecting appropriate camera(s) according to the location of the moving target.

The task for the positioning system, following the calibration of cameras, was assigning the cameras that could detect and locate the target, initially. It required the system to be able to conduct intelligent self-configuration, in terms of automatically identifying the appropriate camera(s), according to the location of the target, while it was moving. The criterion of activating the camera was that the target appeared in the field of vision of the camera. Figure 3a schematically illustrates a target (*P*), six cameras and their corresponding field of visions. If the current location of the target was not in the camera’s field of vision, the camera was in its standby mode, such as the cameras numbered 1, 2, 15, and 16 in Figure 3a. If the current location of the target was in the rectangular field of vision, the camera was activated and in its operative mode. For example, the two cameras, numbered 13 and 14, shown in Figure 3a:

Practically, the field of vision projected on the work plane (*XY* plane) was a rectangular area, because of the geometry of the CCD (charge-coupled device)-sensors matrix of cameras. This rectangular visual region was demarcated by four feature points, i.e., four pixels on the corners of the CCD matrix. Their coordinates were set to (0, 0), (0, 768), (1024, 768), and (1024, 0).

**Figure 4:**
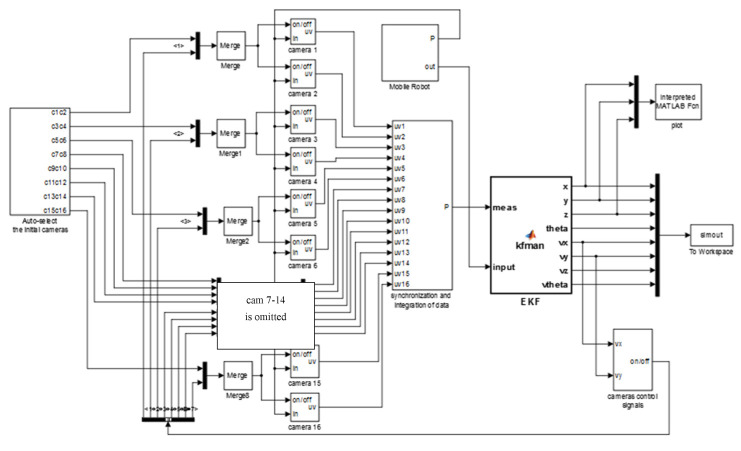
A total of 16 cameras provide the coordinates, in pixels, to subsequent modules. Synchronization and integration of data integrates the datum from cameras, calculates the coordinates of *p*_1_, *p*_2_, *p_3_*, and calculates the position and orientation of the mobile robot (*P*). The EKF estimates the position and velocity ($x,y,z,\theta ,v_{x},{\;v}_{y},v_{z},\dot{\theta }$) of the mobile robot. According to $v_{x},{\;v}_{y}$, the camera control signals model the output signals, to let cameras work (or sleep).

Aiming to ensure that every point in the default trajectory for the moving target was surveilled by at least two cameras, overlap of the rectangular visual regions was set up during switching cameras, when the target was moving around the boundaries of the visual areas. Figure 3b illustrates the situation whereby the target (*P*) moved from the region covered by cameras 13 and 14 to the region covered by cameras 15 and 16. In this transitive approach, it took a little time to switch cameras and process data, requiring the region to be visually covered by at least four cameras, including the two current cameras and two cameras which were to be activated next. To precisely determine the appropriate moment to proceed with the camera switching approach, the parameter *θ*, the angle between *X* axis and the line *OP* (see Figure 3b), was adopted in this work, and acted as the numerical criterion for selecting appropriate camera(s), according to the location of the moving target. To carry out such camera-switching processes in this work, an EKF predictor was utilized. It was able to identify the camera number within the visual region from which the target was about to leave, or into which the target was about to enter. The detailed structure of the EKF predictor was built up in Matlab/Simulink. A model was performed in Matlab, according to velocity ($v_{x},{\;v}_{y}$) predicted by EKF, as in Figure 4:

In the EKF model, the noise of the input and estimated output were pre-set, as follows:

R = diag([0.005^2 0.005^2 0.005^2 0.0005^2]);

Q = diag([0.01 0.01 0.01 0.001 0.01 0.01 0.01 0.001 0.01 0.01 0.01 0.001 0.01 0.01 0.01 0.001]);

The algorithm code for automatically selecting the cameras’ output-controlling signals is listed below:

if theta<theta1

then c13,c14 working, the other sleeping

if theta >= theta1&theta <theta2

then c13,c14,c15,c16 working, the other sleeping

if theta >= theta2&theta <theta3

then c15,c16 working, c13,c14 and the other sleeping

if theta >= theta3&theta <theta4

then c15,c16, c1,c2 working, the other sleeping

……etc.

### 3.3. Self-Configuration for Camera Network

**Figure 5:**
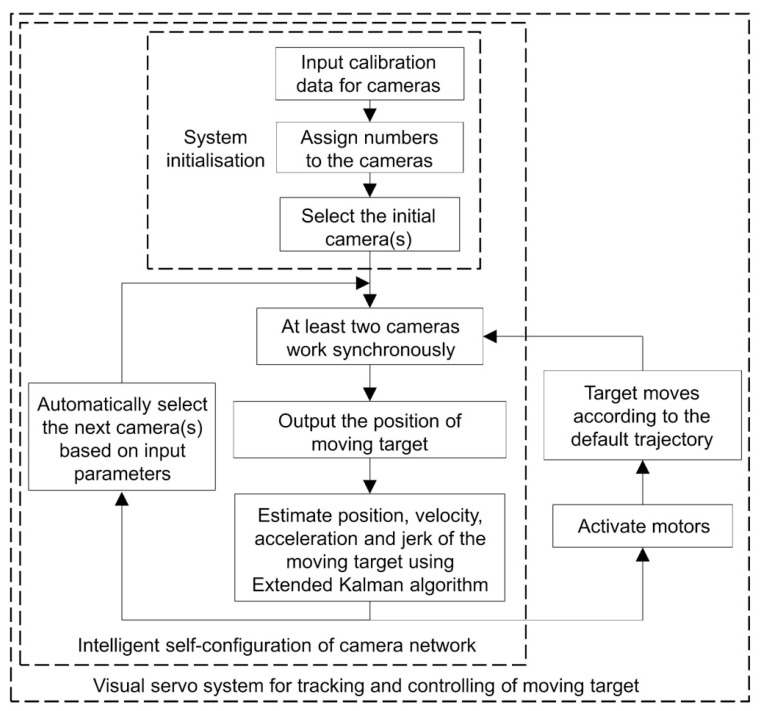
Flow chart of the logical procedures enabling intelligent navigation of the target.

The visual positioning system developed in this work is capable of tracking, as well as controlling, the moving target, thereby enabling intelligent navigation of the target. The procedures for understanding the internal logical steps are illustrated in Figure 5, as a flow chart. The procedures can be divided into three modules, i.e., system initialization, intelligent self-configuration of the camera network, and a visual servo system for tracking and controlling the moving target.

The system initialization involves three tasks: calibrating the cameras, individually assigning numbers to the cameras, and determining the activation of the cameras, based on the initial position of the target. The second module, which is the core of the visual positioning system, enables the intelligent self-configuration. The function of this module is primarily based on the application of the extended Kalman Filter (EKF) algorithm. The procedure for automatically selecting cameras in this module can be summarized by the following step-by-step instructions: (1) first, cameras provide the acquired visual information about the moving target, in terms of pixels, to the subsequent modules; (2) synchronization and integration of data from cameras are then conducted, to calculate the coordinates of point *p*_1_, *p*_2_, and *p_3_*, thereby locating the current position and orientation of the moving target, i.e., the filament winder, in this work; (3) the EKF operation is then used to estimate the velocity, acceleration, and jerk of the moving target; (4) predictions for the route of the moving target can then be achieved, based on the information given for the velocity, acceleration, and jerk, thereby determining the status of the cameras, from one of three action states: be activated, remain unchanged, or get deactivated in the next step. This critical signal is output from the EKF and represents the feedback loop for controlling the cameras. The process loop then starts again from step 1.

Following the EKF calculation, the output signals for both selecting the cameras and controlling the moving target can be obtained from the system. Such output controlling signals consisting of current velocity (speed and direction), acceleration and steering angle, ensure that the system has full control of the moving target and is capable of navigating the target so that it moves exactly following the designated route.

## 4. Results

### 4.1. Experimental Observations

**Figure 6:**
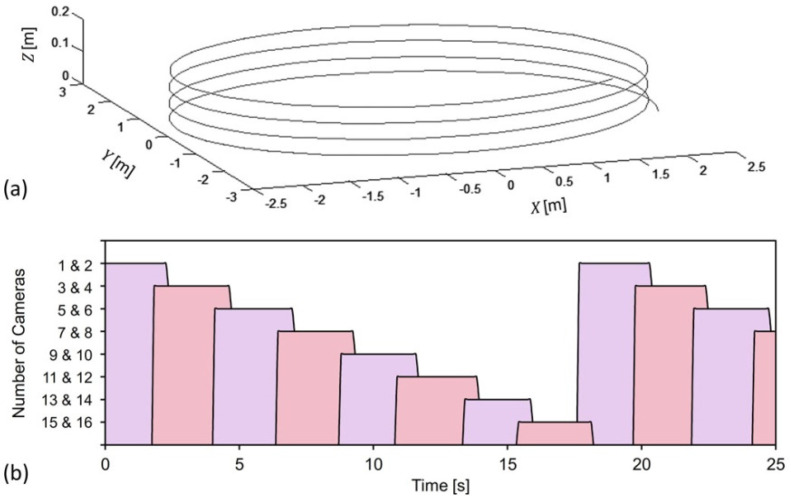
(**a**) The trajectory of fibers on the mandrel, which is constructed by the filament winder in this work. (**b**) Experimentally obtained time schedule for cameras in their alternate operation mode.

The trajectory of the fibers was digitalized and displayed simultaneously with the experimental winding process, which was done by the filament winder. The digitalized trajectory is illustrated in Figure 6a. The trajectory is observed as a helical three-dimensional curve with uniform diameter of 4 m. The distance between two circles in the trajectory is entirely dependent on the rising speed of the mandrel when the filament winder moves along the circular route. The dynamic coefficients of the filament winder, in terms of position and velocity, were obtained as experimental observation data, in real time. Acceleration and jerk of the filament winder were then worked out and output, by the module of the EKF predictor, as the numerical calculated data.

As introduced in the methodology section, to guarantee the locating accuracy in the moving target, the filament winder, at any position in the designated route, should be covered by at least two cameras. According to the arrangement of the cameras and their corresponding assigned numbers, as shown in Figure 2b, the cameras can be grouped with those nearest. For instance, cameras no.1 and no. 2 are always in synchronized working status, because these two cameras are in the same radius of the circular working area (see Figure 2b).

The time schedule of the 16 cameras, when positioning the filament winder, is displayed in Figure 6b. Such a time schedule, which represents the intelligent self-configuration of the camera network, is entirely dependent upon the speed and the designated route of the filament winder. As is displayed in Figure 6b, grouped cameras are activated and deactivated in turn. Cameras no. 1 and 2 were first activated, and deactivated in the time of 2.5 s, approximately. Before the deactivation of cameras no. 1 and 2, cameras no. 3 and 4 were activated in the time of 2 s, approximately. Evidently, there were four cameras in their working status during the time of 2 to 2.5 s. This corresponds to the transitive approach of the cameras, i.e., the camera switching progress, and indicates that the filament winder is concurrently in the overlap area of the rectangular visual regions of cameras no. 1, 2, 3, and 4, during the time of 2~2.5 s. Because the speed of the filament winder is constant, the length of the activated time for all groups of cameras is the same, at the value of approximately 3 s. After the activation of cameras no. 15 and 16, cameras no. 1 and 2 were activated again, at the time of 17.5 s. This suggests that the filament winder had already completed a full circular trajectory, and retuned back to the place where the designated route started. Thus, the period of the movement can be read as taking17.94 s for the filament winder to go all the way around the mandrel. Duplications of such moving periods, round and round, enable continuous winding of the fibers on the mandrel.

**Figure 7:**
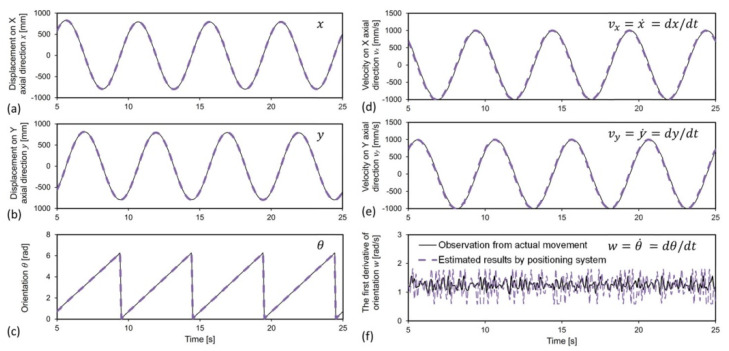
Comparison of parameters from actual observation (black solid lines) and that estimated by the visual positioning system (purple dashed lines), including the positional parameters *x*, *y*, *θ*, and the kinetic parameters *v_x_, v_y_*, and *w*, which are defined in Equation (6).

Figure 7 compares real data, from actual observation (black solid lines), with measured data, estimated from the visual positioning system (purple dashed lines), in terms of positional parameters (*x*, *y*, *θ*) and kinetic parameters (*v_x_, v_y_, w*). Displacement of the moving exhibits target, on *X* axis or *Y* axis, a simple harmonic behavior, with respect to time, because of the circular designated route for the filament winder. As observed in Figure 7a–c, the purple dashed lines and the black solid lines are identical. This qualitatively indicates that the visual positioning system developed in this work can precisely locate the moving target. However, for the linear velocities (*v_x_*, *v_y_*), there is a slight offset between the curves, which can be seen in Figure 7d,e. The purple dashed lines appear slightly ahead of the black solid lines for the whole period of time. Such behavior prediction is primarily owing to the application of the extended Kalman filter (EKF) in the cameras’ self-configuration stage. Specifically, the measured velocity from the EKF predictor, at every point, is numerically calculated by linear prediction, based on the tendency of the former two points, thereby inheriting the mathematical characteristics of the trend before the current point. Such technique was also used in the literature [29] for plotting the tangent modulus from the stress-strain correlations. Thus, the estimated results calculated by the visual positioning system are theoretically ahead of the actual observed results, which are displayed in Figure 7d,e. The angular speed of the moving target in terms of *w* has been depicted in Figure 7f. Significant divergence can be observed between the real data and the measured data. The reason for such disparity is similar to the cause of the offset between the real data and measured data for the parameters of linear velocities (*v_x_*, *v_y_*). Because the prediction of the current angular speed inherits the tendency of the former angular speed, controlling signals from the output of the navigating system lead to an abrupt increase or decrease in the angular speed. As a consequence, overshoot of the predictive curve (see fluctuations of purple curve in Figure 7f), which comprises estimated data points, is likely to occur.

### 4.2. Error Analysis

**Figure 8:**
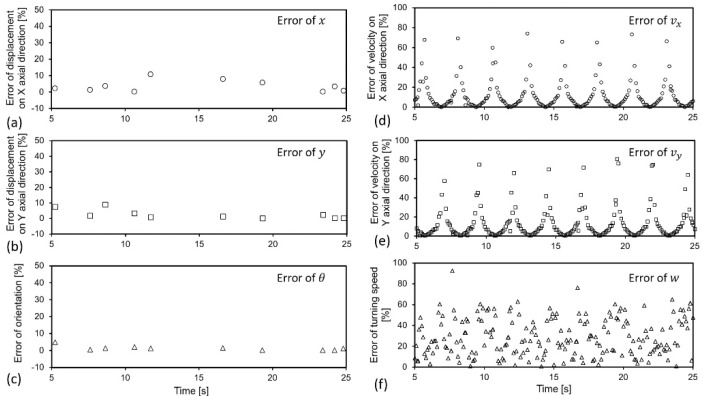
Relative errors plotted versus time for positional parameters (**a**–**c**) and kinetic parameters (**d**–**f**). Errors of positional parameters are kept within 10%, which demonstrates the high-precision locating functions of the visual positioning system.

To quantitatively evaluate the fitness of the real data (actual observation) and the measured data (results estimated by the positioning system), variation of the relative error, in percentage, with time, was calculated and collected in Figure 8, with respect to the positional parameters (*x*, *y*, *θ*) and kinetic parameters (*v_x_*, *v_y_*, *w*). In general, the error for positional parameters (*x*, *y*, *θ*) corresponding to the Figure 8a–c are all within 10%. The averaged errors are calculated as 3.65%, 2.65%, and 1.31%, for the positional parameters *x*, *y*, and *θ*, respectively. These values indicate the high precision of the visual positioning system in obtaining the positional information for the moving target. The averaged errors of the kinetic parameters *v_x_*, *v_y_,* and *w* are calculated as 18.96%, 13.27%, and 29.13%, respectively. These values are slightly larger than that for positional parameters. It may be limited by the resolution of the cameras, or the blurry object boundaries resulting from residual images obtained by the cameras. These residual images or trajectories are very likely to occur when the object moves fast.

Further quantitative evaluations on the fitness between the real data and the measured data have been conducted using the technique of the one-way analysis of variance (ANOVA test). The ANOVA test is often used for judging the fitness between two groups of data [30]. If the calculated value F is smaller (or larger) than the critical value, the ANOVA test confidently indicates that the two groups of data are statistically equal (or not statistically equal). In this work, an ANOVA test with a significance level of *α* = 0.01 was performed. Table 1 lists the results from the ANOVA test on both positional and kinetic parameters. It can be observed that obtained values F are smaller than the critical value, except the data group for angular speed, *w*. Therefore, it shows 99% confidence (1-*α*) that the real data and the measured data for the positional parameters (*x*, *y*, *θ*) and the linear velocities (*v_x_, v_y_*) are statistically equal. Acceptable fitness of real data and measured data has been achieved for these parameters. For the angular speed *w*, the ANOVA test demonstrates that the real data and the measured data are not equal, which implies that the result, in terms of angular speed, obtained from the visual positional system, is not statistically accurate.

## 5. Conclusions

An intelligent, self-configurable visual positioning system based on a camera-network was developed and investigated in this work. The present paper introduces this visual positioning system in relation to a specific industrial application, i.e., tracking the movement of a filament winder. The methodology of locating the filament winder has been elaborated, including the algorithms for enabling calibration of cameras, the automatic selection of cameras, and self-configuration for the camera network. The technique of extended Kalman filter (EKF) was involved in the data processing, aiming to numerically estimate the kinetic coefficients (i.e., velocity, acceleration, and jerk) of the filament winder on the basis of experimentally obtained positioning.

The moving trajectory, time schedule of automatically switching cameras, and time-domain chart of positional parameters (*x*, *y*, and *θ*) and kinetic parameters (*v_x_*, *v_y_*, and *w*) have been experimentally recorded and illustrated. General error analysis and an ANOVA test have been carried out to determine the quality of positioning of the system. Relative errors for positional parameters are all smaller than 10%; relative errors for linear velocities (*v_x_*, *v_y_*) are also kept to an acceptable level, i.e., lower than 20%. The result from the ANOVA test verifies such a conclusion, which suggests that the real data and the measured data for the positional parameters (*x*, *y*, *θ*) and the linear velocities (*v_x_*, *v_y_*) are statistically equal, with 99% confidence.

In conclusion, these results demonstrate that the camera-network-based visual positioning system is capable of locating a moving target with high precision, and predicting the linear velocities of the moving target with acceptable accuracy. It presents the outstanding potential of this visual positioning system to assist in the industry of automation, including wireless intelligent control, high-precision indoor positioning, and navigation.

## Figures and Tables

**Table 1:** Quantitative evaluation of the fitness of real data and measured data, in terms of positional parameters (*x*, *y*, *θ*) and kinetic parameters (*v_x_*, *v_y_*, *w*).

	*x*	*y*	*θ*	*v_x_*	*v_y_*	*w*
*F* *	2.31 × 10^−6^	1.69 × 10^−6^	2.16 × 10^−4^	5.23 × 10^−6^	4.14 × 10^−4^	9.72
*F^CV^* *	6.69	6.69	6.69	6.69	6.69	6.69
Interpretation	*F < F^CV^*	*F < F^CV^*	*F < F^CV^*	*F < F^CV^*	*F < F^CV^*	*F > F^CV^*

* *F* and *F^CV^* are the obtained value and critical value, respectively, from one-way analysis of variance (ANOVA) test.

## Data Availability

The study did not report any data.
